# Characterization of the *Escherichia coli* Virulent Myophage ST32

**DOI:** 10.3390/v10110616

**Published:** 2018-11-07

**Authors:** Honghui Liu, Hany Geagea, Geneviève M. Rousseau, Simon J. Labrie, Denise M. Tremblay, Xinchun Liu, Sylvain Moineau

**Affiliations:** 1 College of Resources and Environment, University of Chinese Academy of Sciences, Beijing 101408, China; honghuiliu@hotmail.com; 2 Département de Biochimie, de Microbiologie, et de Bio-Informatique, Faculté des Sciences et de Génie, Université Laval, Québec City, QC G1V 0A6, Canada; Hany.Geagea.1@ulaval.ca (H.G.); Genevieve.Rousseau@greb.ulaval.ca (G.M.R.); simon.labrie@syntbiolab.com (S.J.L.); Denise.Tremblay@greb.ulaval.ca (D.M.T.); 3 Félix d’Hérelle Reference Center for Bacterial Viruses, Faculté de médecine dentaire, Université Laval, Québec City, QC G1V 0A6, Canada

**Keywords:** *Escherichia coli*, virulent phage, genomic characterization, lytic activity, biocontrol

## Abstract

The virulent phage ST32 that infects the *Escherichia* *coli* strain ST130 was isolated from a wastewater sample in China and analyzed. Morphological observations showed that phage ST32 belongs to the *Myoviridae* family, as it has an icosahedral capsid and long contractile tail. Host range analysis showed that it exhibits a broad range of hosts including non-pathogenic and pathogenic *E. coli* strains. Interestingly, phage ST32 had a much larger burst size when amplified at 20 °C as compared to 30 °C or 37 °C. Its double-stranded DNA genome was sequenced and found to contain 53,092 bp with a GC content of 44.14%. Seventy-nine open reading frames (ORFs) were identified and annotated as well as a tRNA-Arg. Only nineteen ORFs were assigned putative functions. A phylogenetic tree using the large terminase subunit revealed a close relatedness with four unclassified *Myoviridae* phages. A comparative genomic analysis of these phages showed that the *Enterobacteria* phage phiEcoM-GJ1 is the closest relative to ST32 and shares the same new branch in the phylogenetic tree. Still, these two phages share only 47 of 79 ORFs with more than 90% identity. Phage ST32 has unique characteristics that make it a potential biological control agent under specific conditions.

## 1. Introduction

Pathogenic *Escherichia coli* (*E. coli*) is a common zoonotic agent that poses a significant threat to public health and safety. Shiga-toxin-producing *E. coli* (STEC) strains are one of the most important foodborne pathogens [1,2]. The Shiga toxin (Stx) cleaves ribosomal RNA, thereby disrupting protein synthesis and killing the intoxicated epithelial or endothelial cells [3]. STEC infection can result in diseases such as diarrhea, hemorrhagic colitis, and hemolytic-uremic syndrome (HUS) in humans and animals. These diseases are subjected to various pharmaceutical treatments including antibiotics, such as ampicillin, streptomycin, sulfonamides, and oxytetracycline [4,5].

It is well-known that the use of antibiotics can lead to the spread of antibiotic-resistant bacteria in the environment, which poses a risk to human health [6,7,8]. Antimicrobial resistance of *E. coli* is an issue of the utmost importance since it can affect both animals and humans [9]. This bacterial species has a great capacity to accumulate antibiotic resistance genes, mostly through horizontal gene transfer [10,11]. For example, the intensive use of various antibiotics in aquaculture has had significant benefits to the fish industry but it has also led to serious negative effects on the environment, including the emergence of a pool of antibiotic-resistant bacteria and transferable resistance genes [6,12,13,14]. Some of those antibiotic-resistance genes can be transferred horizontally from bacteria in aquatic environments to pathogenic bacteria, affecting land animals and humans [13,14]. Moreover, the transmission of resistant clones and resistance plasmids of *E. coli* from poultry to humans has also been identified [15,16].

Of note, the highest rate of antibiotic-resistance genes was found in *E. coli* strains of a sewage treatment plant that treats both municipal and hospital sewage [17,18,19]. Although wastewater treatment processes reduce the number of bacteria in sewage by up to 99%, *E. coli* cells can still reach the receiving water and contribute to the dissemination of resistant bacteria into the environment [20]. As a result, antimicrobial resistance in *E. coli* is considered one of the major challenges for both humans and animals at a worldwide scale and it needs to be considered as a real public health concern.

Alternative strategies must be developed to reduce the risk associated with the dissemination of antimicrobial resistance and to control the risk of disease transmission. The use of phages as biocontrol agents has received increasing attention recently as a possible alternative or as a complement to antibiotics [21,22,23,24,25,26,27]. For example, bacteriophages have demonstrated efficacy in controlling pathogenic bacterial populations in, among others, poultry meat [28], aquaculture [23], wastewater, and minimally processed, ready-to-eat products and fresh fruits [25,29,30,31]. It can also help to remove bacteria on chicken skin [22] and on dairy cows at different lactation stages [26]. Interestingly, these bacterial viruses can be highly specific to a single bacterial species or to only a few strains within that species, or can productively infect a range of bacterial species [32,33].

In the present study, we used the host pathogenic *E. coli* ST130 (flagellin H21) carrying Shiga toxin (*stx*1, *stx*2) genes to isolate and characterize a new virulent coliphage, named ST32. This phage was isolated from sewage water and possesses appealing characteristics that could be of interest for specific biocontrol purposes.

## 2. Materials and Methods

### 2.1. Bacterial Strain

Escherichia coli ST130 was obtained from the Chinese Center for Disease Control and Prevention (China CDC). This bacterium was used as the phage host.

### 2.2. Phage Isolation and Purification

Phage ST32 was isolated from a wastewater sample of a sewage treatment plant in Beijing, and was propagated and titrated using methods described previously [34]. Samples were filtered with a 0.45 μm sterile PES syringe filter (Sarstedt, Nümbrecht, Germany, catalog number 83.1826), and then, 2.5 mL of the filtered sample and 1 mL of an overnight *E. coli* ST130 culture were added to 7.5 mL of Luria broth (LB) (1% bacto-tryptone, 0.5% bacto-yeast extract, and 1% NaCl) incubated overnight with agitation (200 rpm) at 37 °C. The resulting supernatant was filtered and serially diluted in order to isolate phage plaques using the double layer agar method. Briefly, 100 μL of serially diluted lysate and 100 μL of an overnight *E. coli* culture were added to 4 mL of LB supplemented with 0.75% agar. The inoculated soft agar was then poured into LB plates (1.5% agar). The plates were incubated overnight at 37 °C, and single phage plaques were picked, propagated, and purified three times.

### 2.3. Phage Morphology

Phage ST32 was purified and concentrated by CsCl gradient as described previously [35]. Phage particles were stained with 2% (*w*/*v*) aqueous uranyl acetate on a carbon-coated grid and were observed using a JEM-1230 transmission electron microscope (JEOL, Tokyo, Japan) [36]. Over 10 specimens were observed and used for size determination.

### 2.4. Host Range

The host range of phage ST32 was tested on 73 bacterial strains from different genera, species, and serotypes using the spot test method and a diluted phage lysate. In brief, 200 μL of overnight culture of *E. coli*, *Shigella*, *Salmonella*, or *Citrobacter* was mixed with 3.5 mL of LB containing 0.75% (*w*/*v*) soft agar. The inoculated soft agar was then poured on LB (1.5% (*w*/*v*) agar) plates. Then, serial dilutions of phage lysate were made in buffer (50 mM Tris−HCl at pH 7.5, 100 mM NaCl, and 8 mM MgSO_4_). Five microliters of various serial dilutions (10^0^, 10^−2^, 10^−4^ and 10^−6^) was spotted on the top agar. After overnight incubation at 37 °C, phage plaques or lysis zones were recorded.

Moreover, the propagation of phage ST32 on non-pathogenic host strains (*E. coli* HER1036, HER1155, HER1222, HER1315, HER1375, and HER1536) was compared to that of the pathogenic *E. coli* ST130 strain. In brief, the strains were grown at 37 °C in LB medium until an optical density at 600 nm (OD) of 0.25. Then, approximately 10^6^ PFU·mL^−1^ of phage ST32 was added. The phage-infected cultures were incubated with agitation at 37 °C until complete bacterial lysis was achieved. The phage lysate was centrifuged to remove cell debris, and the supernatant was filtered using a 0.45 μm syringe filter. Then, the phage lysates were serially diluted in buffer and titered by spot test as described above. Of note, the pathogenic *E. coli* ST130 strain was used for phage titration after propagation.

### 2.5. One-Step Growth Curve Assay

The influence of the incubation temperature on phage ST32 plaque formation was investigated by spot test as described above. Following the spot test assay, the plates were incubated at various temperatures (ranging from 10 to 42 °C).

One-step growth curve assays were also performed in triplicate. Briefly, phages were mixed with 2 mL of a mid-exponential phase culture of *E. coli* ST130 (OD of 0.8) with a starting multiplicity of infection (MOI) of 0.05. ST32 phages were allowed to adsorb to *E. coli* ST130 cells for 5 min at various temperatures (20, 30, or 37 °C), and then the mixture was centrifuged for 1 min at 16,000× *g*. The pellet was resuspended, diluted, and added to 10 mL of LB. This suspension was incubated at three different temperatures (20, 30, or 37 °C) without agitation, and samples were taken to test the phage titers. The phage titer of each sample was determined using the double layer agar method. All plates were incubated overnight at 30 °C. The burst size was calculated by subtracting the initial titer from the final titer and then dividing by the initial titer. The latent phase corresponded to the middle of the exponential phase of the curve [37]. The data were analyzed under a one-way analysis of variance (ANOVA) followed by a Tukey test to correct the *p*-values for the multiple comparisons. Significant differences were reported at an alpha level of 1%.

### 2.6. E. coli ST130 Growth

*E. coli* ST130 growth was also determined at various temperatures using OD and recorded in triplicate. In brief, 200 μL of ST130 overnight culture was added to 5 mL of LB medium. Then, inoculated samples were incubated with agitation (200 rpm) at 20, 30, and 37 °C. The OD was measured at intervals of 30 min.

### 2.7. Sequencing and Analysis

Phage DNA was extracted as described elsewhere [38]. DNA was sequenced using the Illumina Hiseq (PE250) platform at Beijing Fixgene Tech Co., Ltd. (Beijing, China). More than 5000-fold coverage of the phage genome was generated. The paired-end reads were assembled using ABySS v. 1.3.6. Open reading frames (ORFs) were predicted using PHASTER [39]. The identified ORFs were confirmed with GeneMark.hmm prokaryotic (http://exon.gatech.edu/GeneMark/gmhmmp.cgi) and ORF Finder (https://www.ncbi.nlm.nih.gov/orffinder/). ORFs were considered candidates for evaluation when they encoded 45 or more amino acids (aa) and possessed both a conserved Shine–Dalgarno sequence (5′-AGGAGGU-3′) and a start codon (AUG, UUG, or GUG). BLASTp was used to identify the putative functions of the proteins. Hits were considered valid when the E-value was lower than 10^−3^. The percent identity between proteins was calculated by dividing the number of identical residues by the size of the smallest protein. The theoretical molecular weights (MW) and isoelectric points (pI) of the proteins were obtained using tools available on the ExPASy webpage (http://web.expasy.org/compute_pi/). The bioinformatic tool tRNAscan-SE (http://lowelab.ucsc.edu//tRNAscan-SE/) was used for tRNA detection.

### 2.8. Terminase Tree

A phylogenetic tree was generated based on the large terminase subunit amino acid sequences of phage ST32 and multiple phages available in databases sharing sequence identity. The corresponding phage protein sequences were retrieved from GenBank (https://www.ncbi.nlm.nih.gov/). In constructing the terminase phylogenetic tree, these sequences were aligned with MAFFT [40] using the E-INS-i alignment algorithm. Thereafter, MAFFT-profile alignment was processed, as previously described [41], in order to generate the tree. Briefly, ProtTest 3.2 was applied to find an appropriate model of amino acid substitution and was implemented in PhyML 3.0 to calculate a maximum likelihood tree. Finally, the Shimodaira–Hasegawa-like procedure was used to determine the branch support values and the Newick utility package was used to render the trees.

### 2.9. Nucleotide Sequence Accession Number

The complete genome sequence of phage ST32 was deposited in GenBank under the accession number MF044458.2.

## 3. Results and Discussion

### 3.1. Phage Morphology

**Figure 1:**
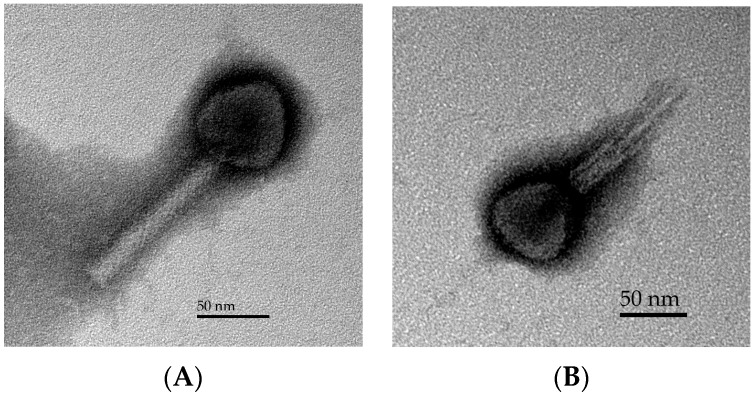
Transmission electron micrograph of phage ST32 with uncontracted (**A**) and contracted (**B**) tails.

The morphological characteristics of phage ST32 were examined by transmission electron microscopy. Electron micrographs (Figure 1) showed that phage ST32 has an icosahedral capsid with an apex diameter of 64 ± 6 nm and a long contractile tail with a length of 132 ± 9 nm. These morphological features [42] indicate that phage ST32 belongs to the *Caudovirales* order and the *Myoviridae* family.

### 3.2. Host Range

Currently, phages are tested for biocontrol purposes against *E. coli* strains that may cause infections [43,44] or used as indicators of coliform contamination [45]. The host range plays a key role in the selection of any given phage for therapy or biocontrol purposes, as a broad host range phage is likely to kill multiple strains of a given bacterial species and maybe even beyond the species or genus levels for enterophages [43,46].

To this end, the host range of phage ST32 was evaluated on 73 bacterial strains obtained from the Félix d’Hérelle Reference Center for Bacterial Viruses (Table 1). Phage ST32 was able to infect 10 strains (14%), including four pathogenic and six non-pathogenic strains. Pathogenic strains infected by phage ST32 included four *E. coli* strains of multiple serotypes. In order to reduce the risk of possible harmful substances from the pathogenic host strain in phage lysate, we evaluated the ability of phage ST32 to propagate on its sensitive, non-pathogenic host strains (++++; Table 1). The results showed that phage ST32 was propagated to a high titre (10^9^ PFU/mL) when using five (*E. coli* HER1036, HER1222, HER1315, HER1375 and HER1536) out of six of these strains.

Based on the above, phage ST32 has a broad host range, infecting both pathogenic and non-pathogenic *E. coli* strains. These features led us to consider phage ST32 to be a potential biocontrol agent rather than a therapeutic agent. In order to use phage ST32 as a biocontrol agent, we further studied the influence of temperature on its lytic activity as well as on the growth of *E. coli* host strain ST130.

### 3.3. One-Step Growth Curve

The influence of temperature on plaque formation was first analyzed by spot test at 10, 20, 30, 37, and 42 °C. The results showed that phage ST32 produced clear plaques at dilutions of 10^−1^ to 10^−7^ when plates were incubated at 10, 20, 30, and 37 °C. Turbid plaques were seen but only at 42 °C. A one-step growth curve was conducted at 20, 30, and 37 °C to determine its latent period and burst size at these temperatures. Moreover, the growth of the bacterial host strain followed under the same conditions.

**Figure 2:**
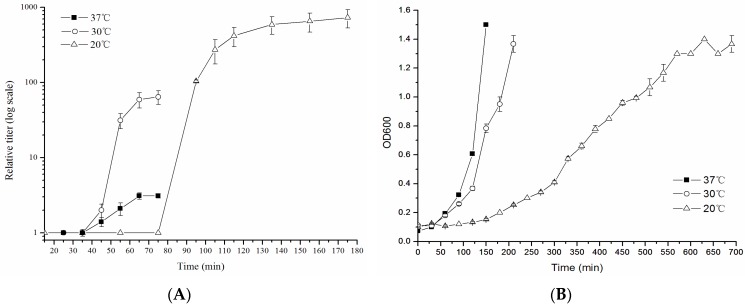
Lytic activity of phage ST32 at various temperatures. (**A**) One-step growth curve of phage ST32 at 20 °C, 30 °C, and 37 °C; (**B**) The growth of *E. coli* ST130 strain at 20 °C, 30 °C, and 37 °C.

As indicated by the results of the one-step growth curve experiments (Figure 2a), the burst size of phage ST32 was very low at 37 °C, to the extent that only 2 ± 0.1 new virions were released per infected cell with an estimated latent period of 55 ± 6 minutes. When the phage-infected cells were incubated at 30 °C, the average burst size of phage ST32 increased to 64 ± 30 new virions per infected cell, and the latent period remained the same (54 ± 2 min). Interestingly, the burst size of phage ST32 was significantly higher when the infected cells were incubated at 20 °C with an average of 602 ± 159 new virions being released per infected cell. Conversely, the latent period increased to approximately 102 ± 10 min. Of note, the growth of the *E. coli* ST130 host strain was much faster at 30 °C and 37 °C compared to that at 20 °C (Figure 2B). Nonetheless, phage ST32 could still kill its host at these temperatures.

Phage ST32 is evidently part of a low-temperature (LT) phage group with an optimum burst at 20 °C [48]. Of note, this phage was isolated from a wastewater sample of a sewage treatment plant in Beijing that has a temperature of about 20 °C. Therefore, it appears to be adapted to replicate at such ambient-like temperatures. These features make this phage a potential agent for the biocontrol of *E. coli*. For instance, it could be used to control pathogenic bacteria present in wastewater where physical conditions, such as temperature, are optimal for its lytic activity. Moreover, it may provide an effective intervention against foodborne pathogens and spoilage bacteria in minimally processed, ready-to-eat products and fresh fruits [29,30,31]. It could also help to remove bacteria from poultry meat that are often found to be contaminated with potentially pathogenic micro-organisms [28]. In order to support its potential as a biocontrol agent, we further characterized phage ST32 at the genomic and phylogenetic levels.

### 3.4. Genomic Features of Phage ST32

The genome sequence of phage ST32 consists of a double-stranded DNA molecule of 53,092 bp with a GC content of 44.14% as well as 79 open reading frames (ORFs) and a tRNA (Table 2). The tRNA-Arg of 95 bp (from 15,909 bp to 16,003 bp), without an intron, found in the genome of phage ST32, shares 99% identity with phage phiEcoM-GJ1 [49]. tRNA-Arg is often found in phage genomes [50]. The 79 ORFs have the same transcriptional orientation, and ATG is the most common initiation codon (81.0%), followed by GTG (11.4%) and TTG (7.6%).

Based on the BLASTp analyses, 19 of the 79 ORFs (24.1%) were assigned a putative function, including lysis, capsid, and tail morphogenesis as well as transcription and DNA replication. The functions of the remaining sixty putative ORFs remained unknown, and they were annotated as hypothetical proteins. Besides the predicted protein functions, Table 2 shows the predicted size, the genomic position, the transcriptional orientation, and the closest phage protein homolog. In several cases, protein homologies were with proteins of phages belonging to the *Podoviridae* or *Myoviridae* families. The best matches for a large portion of these ORFs were with proteins of the *Enterobacteria* phage phiEcoM-GJ1 belonging to the *Myoviridae* family [49]. Thereafter, phylogenetic trees were constructed for further investigation of the relatedness of phage ST32 to other phages.

### 3.5. Phylogeny of Phage ST32

**Figure 3:**
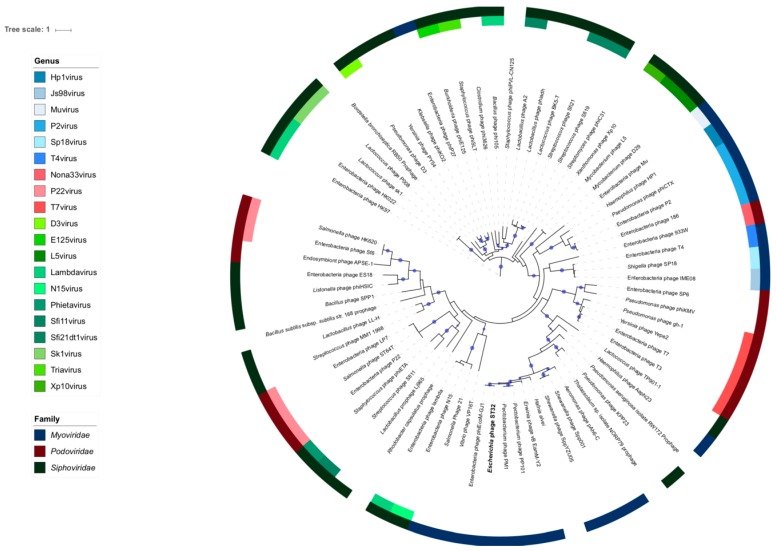
Phylogenetic tree based on the amino acid sequences of the large terminase subunit (ORF51) of phage ST32 and the phages available in databases sharing sequence identity. The corresponding phage protein sequences were retrieved from GenBank (https://www.ncbi.nlm.nih.gov/). The colors in the internal and external circular layers categorize phages, genera, and families, respectively. When the genera or the family of a phage is not indicated, it means that it was not available in the database or in the associated publication. Branches with branch support values greater than 90% are marked with a blue dot. The size of the dot is directly proportional to the branch support value.

The conserved sequence of the large terminase subunit (ORF51) has been used previously to study the phylogeny of numerous phages [41,42]. As an ATP-driven protein motor, the phage terminase is generally a hetero-oligomer composed of two subunits (small and large) that translocates the phage genome into the preformed capsid. The large subunit usually possesses endonucleolytic and ATPase activities [51,52]. A phylogeny tree, based on the amino acid sequences of the large terminase subunit (ORF51), was constructed to examine the evolutionary relationships between phage ST32 and other phage genomes (Figure 3). The phylogeny tree supported the finding that phage ST32 belongs to the Myoviridae family. Moreover, phage ST32 was on the same branch as phage phiEcoM-GJ1 (EF460875.1), indicating a close relatedness between these two phages and suggesting that they belong to the same new cluster. Interestingly, phiEcoM-GJ1 phage currently belongs to an unclassified genus of the *Myoviridae* family [49]. Moreover, the tree indicated that the closest evolutionary relatives to both phages were the *Pectobacterium* virulent phages PM1 [53] and PP101 and the *Erwinia* virulent phage vB_EamM-Y2 [54]. This relatedness between the PM1, vB_EamM-Y2, and phiEcoM-GJ1 phages was revealed in a previous study [53].

Thereafter, we compared the percent identity between the genome sequences of these five phages. Our results showed that the percentage of nucleotide sequence identity between phages in the same branch was relatively high compared to phages in different branches. For example, the percent identity between phages ST32 and PM1 did not exceed 36% compared to 84.5% between the two *Pectobacterium* phages PM1 and PP101.

### 3.6. Comparative Genomic Analysis

**Figure 4:**
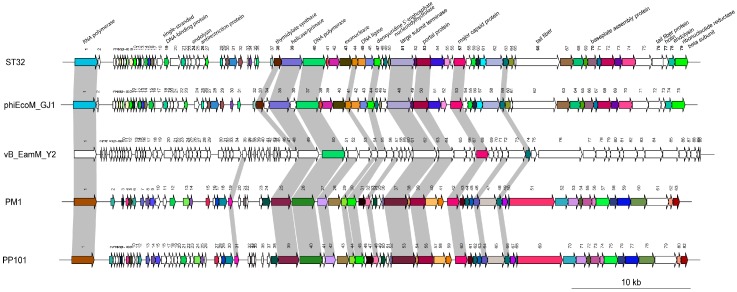
Schematic representation of the genomic organization of phage ST32 compared to phages phiEcoM-GJ1, vB_EamM-Y2, PM1, and PP101. Each line represents a different phage genome and each arrow represents an ORF. Arrows of the same color indicate ORFs that share more than 80% identity. White arrows indicate that the identity is less than 80% or there is no homologous putative protein. Gray shading indicates vB_EamM-Y2 phage ORFs sharing more than 70% with that of other aligned phages.

The genomic sequences of the ST32, phiEcoM-GJ1, PM1, PP101, and vB_EamM-Y2 phages were further analyzed, compared, and aligned using the deduced amino acid sequences of all of the ORFs. A comparative analysis showed that when using a cut-off of 80% identity, phage ST32 shares 54 proteins with phage phiEcoM-GJ1, while the *Pectobacterium* phages PM1 and PP101 share 53 proteins. On the other hand, at the same cut-off, the *Erwinia* phage vB_EamM-Y2 shares only three proteins with the other four phages (Figure 4). Notably, at 70% identity, this number went up to 14 proteins, as indicated by the gray shading in Figure 4.

Interestingly, with more than 60% identity, 31 proteins were shown to be shared by the four phages ST32, phiEcoM-GJ1, PM1, and PP101. Based on this comparative analysis, these five phages can be separated into three distinct groups, which is consistent with their three-branch division in the phylogenetic tree (Figure 3). Based on the close relatedness between phages ST32 and phiEcoM-GJ1 shown in the above analysis, we compared them further.

The genomic organization of phage ST32 compared to phage phiEcoM-GJ1 (Figure 4) showed that all genes from both phage genomes have the same transcription orientation (5′ to 3′ from left to right in the figure). Moreover, 47 of 79 ORFs share more than 90% identity, of which eight (ORF42, ORF49, ORF50, ORF52, ORF60, ORF64, ORF68 and ORF69) are 100% identical (Table 2). The latter are proteins with hypothetical functions. Interestingly, six of these eight ORFs are found in very few phage genomes available in databases [49,53], including the ones closely related to phage ST32 that were used for the genomic comparison in Figure 4.

The global analysis of both phage genomes showed that they are organized into functional clusters to which different roles can be assigned. First, both phages share a cluster of a high number of small genes at the beginning of the genome (starting from ORF2), reminiscent of those on of T4 coliphages which are involved in host takeover [42,49,55] (Figure 4). Most of the phage ST32 ORFs in this cluster share less than 90% identity with those of phage phiEcoM-GJ1 (Table 2). Then, downstream of the genome, several putative replication-related genes were identified, encoding a single-stranded DNA-binding protein (ORF19), thymidylate synthase (ORF38), helicase/primase (ORF39), DNA polymerase (ORF40), 5′-3′ exonuclease (ORF43), DNA ligase (ORF45), deoxyuridine 5′-triphosphate nucleotidylhydrolase (ORF47), and ribonucleotide reductase beta subunit (ORF79). In addition to the replication-related genes, the last ORFs in the genome of phages ST32 and phiEcoM-GJ1 encode a ribonucleotide reductase beta subunit. In this regard, it is interesting to note that the ORF1 of both phages encodes a single-subunit RNA polymerase which is a feature of phages of the T7 group of the *Podoviridae* [49]. These transcription-related ORFs share more than 90% identity (Table 2). Then, downstream of the replication-related genes, we identified a cluster of DNA packaging, capsid, and tail morphogenesis conserved genes sharing more than 90% identity, except for two ORFs, ORF66 and ORF76, encoding for two putative tail fiber proteins and sharing 76% and 72% identity, respectively.

Finally, further main differences were identified between the two phages. For example, three ORFs were only found throughout the genome of phage phiEcoM-GJ1, encoding for three putative HNH endonucleases (ORF34^phiEcoM-GJ1^, ORF36^phiEcoM-GJ1^, and ORF47^phiEcoM-GJ1^) [49]. Moreover, five additional ORFs (ORF17, ORF18, ORF33, ORF35, and ORF56) encoding proteins with unknown functions were found in the genome of phage ST32 but not in that of phage phiEcoM-GJ1. Interestingly, the best match for one (ORF56) of these five ORFs was with that of the *Erwinia* phage vB_EamM-Y2, which is closely related to phage ST32, as shown in the phylogenetic tree (Figure 3).

## 4. Conclusions

In this study, the virulent phage ST32 was isolated from wastewater using the pathogenic host *E. coli* ST130. Morphological and genomic characterization showed that phage ST32 belongs to the *Myoviridae* family. Host range analysis showed that it can infect a broad range of hosts including non-pathogenic and pathogenic bacteria. Moreover, phage ST32 has a very high burst size at 20 °C which is far from the optimal growth of its host. Phylogenetic analysis, based on the large terminase subunit (ORF51), revealed a close relatedness with the *Enterobacteria* phage phiEcoM-GJ1 belonging to an unclassified genus of the *Myoviridae* family. Interestingly, both phages are part of a new branch in the phylogeny. Moreover, neighboring branches carry unclassified *Myoviridae* relatives, among others, the *Pectobacterium* phages PM1 and PP101 and the *Erwinia* phage vB_EamM-Y2. A comparative genomic analysis of the five phages based on nucleotide and amino acid sequences, showed that phage phiEcoM-GJ1 is by far the closest relative to phage ST32. A more detailed genomic comparison between these two phages showed that 47 of 79 ORFs in the phage ST32 genome have more than 90% identity with the phage phiEcoM-GJ1. Many of these ORFs had few homologs in databases. Some striking differences were detected, including the absence of three putative HNH endonucleases of phiEcoM-GJ1 ORFs in phage ST32. On the other hand, five additional ORFs with unknown functions were detected in the phage ST32 genome. Taken together, the newly characterized phage ST32 has appealing and unique characteristics that make it a potential biological control agent under specific conditions.

## Figures and Tables

**Table 1:** The host range of phage ST32.

Non-Pathogen			Pathogen				
Genus/Species/Subspecies of the Host Strain	# HER	Name of the Host Strain	ΦST32	Genus/Species/Subspecies of the Host Strain	# HER	Name of the Host Strain	ΦST32
*Escherichia coli*	1022	O44:K74 MUL-B37.2	−	*Escherichia coli*	1176	N/A	+++
*Escherichia coli*	1024	B (11303)	+	*Escherichia coli*	1255	O157:H7 C-8299-83	−
*Escherichia coli*	1025	K12 C600 (λ)	+	*Escherichia coli*	1256	O157:H7 E318	−
*Escherichia coli*	1036	C (13706)	++++	*Escherichia coli*	1257	O157:H7 A7793-B1	−
*Escherichia coli*	1037	K12S	+	*Escherichia coli*	1258	O157:H7 C-8300-83	−
*Escherichia coli*	1040	K12 (λ) Lederberg	+	*Escherichia coli*	1259	O157:H7 C-7685-84	−
*Escherichia coli*	1077	W3350	+	*Escherichia coli*	1260	O157:H7 CL40	−
*Escherichia coli*	1128	MUL-B70.1	−	*Escherichia coli*	1261	O157:H7 C-7111-85	−
*Escherichia coli*	1129	O86:B7 MUL-B3.1	−	*Escherichia coli*	1262	O157:H7 B1190-1	−
*Escherichia coli*	1139	K12 65	+	*Escherichia coli*	1263	O157:H7 B1328-C10	−
*Escherichia coli*	1144	K12S Lederberg	−	*Escherichia coli*	1264	O157:H7 A8188-B3	−
*Escherichia coli*	1155	K1	++++	*Escherichia coli*	1265	O157:H7 C7420-85	−
*Escherichia coli*	1213	JE-1 (N3)	−	*Escherichia coli*	1266	O157:H7 3283	−
*Escherichia coli*	1217	JE-2(R62Rpilc)	+	*Escherichia coli*	1267	O157:H7 C-7140-85	−
*Escherichia coli*	1218	J53(RIP69)	−	*Escherichia coli*	1268	O157:H7 5896	−
*Escherichia coli*	1219	K12 J62-1(R997)	−	*Escherichia coli*	1269	O157:H7 C-7142-85	−
*Escherichia coli*	1221	K12 J53-1(R15)	+	*Escherichia coli*	1270	O157:H7 C-91-84	−
*Escherichia coli*	1222	JE-1 (RA1::TN5Sqr)	++++	*Escherichia coli*		H21 ST130	++++
*Escherichia coli*	1240	J62-1 (R27::TN7)	+	*Escherichia coli*		O165:H8 ST120	−
*Escherichia coli*	1252	40	+	*Escherichia coli*		O8:H16 ST110	++
*Escherichia coli*	1253	HM 8305	−	*Escherichia coli*		H8 ST100	++
*Escherichia coli*	1271	K12 C600 (H-19J)	+	*Escherichia coli*		O153:H12 BW	−
*Escherichia coli*	1275	K12 C600	+	*Shigella sonnei*	1043	Y6R	+
*Escherichia coli*	1290	CSH39	−	*Shigella* dysenteriae	1031	aSH	−
*Escherichia coli*	1299	K12 C600 (933-J)	+	*Shigella* dysenteriae	1020	SH(P2)	−
*Escherichia coli*	1315	F492 (O8:K27-:H-)	++++	*Salmonella* paratyphi	1045	B type 1	−
*Escherichia coli*	1337	O103 2929	+	*Salmonella typhi*	1038	ViA subtype Tananarive	−
*Escherichia coli*	1366	K12 MC4100	+	*Citrobacter freundii*	1518	CF3	−
*Escherichia coli*	1374	E69 O9:K30:H12	−	*Citrobacter freundii*		CF4	−
*Escherichia coli*	1375	CWG 1028	++++	*Citrobacter freundii*	1516	CF5	−
*Escherichia coli*	1382	Ymel mel-1 supF58	+	*Citrobacter freundii*		CF7	−
*Escherichia coli*	1383	Ymel (HK97)	+	*Citrobacter freundii*		CF8	−
*Escherichia coli*	1392	0103 GVs	−	*Citrobacter freundii*		Sa1	−
*Escherichia coli*	1393	Rougier	−	*Citrobacter freundii*		Sa6	−
*Escherichia coli*	1445	TC4	−	*Citrobacter freundii*		Sa59	−
*Escherichia coli*	1446	MB4	−				
*Escherichia coli*	1462	C-3000	+				
*Escherichia coli*	1536	SlyD	++++				

Notes: (−) Do not infect; (+) lysis zone at dilution 10^0^ or “lysis from without [47]”; (++) infect at dilutions of 10^0^ to 10^−2^; (+++) infect at dilutions of 10^0^ to 10^−4^; (++++) infect at dilutions of 10^0^ to 10^−6^.

**Table 2:** Features of the open reading frames (ORFs) of phage ST32.

ORF	Strand	Start (pb)	End (pb)	Size (aa)	MW (kDa)	pI	SD Sequence (AGGAGGU) ^a^	Predicted Protein Function	BLAST (Extent, % aa Identity) ^b^	Aligned Protein Size (aa)	E Value	Accession Number
1	+	673	2613	646	72.7	6.09	TGGAGACttacaa**ATG**	RNA polymerase	gp01 [*Enterobacteria* phage phiEcoM-GJ1] (598/646; 93%)	648	0	YP_001595396.1
2	+	2640	2846	68	7.89	4.51	AGGATGGcattag**TTG**		gp02 [*Enterobacteria* phage phiEcoM-GJ1] (48/55; 87%)	55	1 × 10^−17^	YP_001595397.1
3	+	3959	4177	72	4.14	8.15	AGGAGAAtaaa**ATG**		hypothetical protein [Klebsiella phage KP8] (23/73; 32%)	71	8 × 10^−4^	AVJ48916.1
4	+	4217	4453	78	8.9	9.63	CGGAGAGcagaa**ATG**		gp04 [*Enterobacteria* phage phiEcoM-GJ1] (49/76; 64%)	76	8 × 10^−23^	YP_001595399.1
5	+	4456	4644	62	7.5	4.53	ACGAGGTtaatc**ATG**		gp05 [*Enterobacteria* phage phiEcoM-GJ1] (61/62; 98%)	62	1 × 10^−37^	YP_001595400.1
6	+	4641	4835	64	7.3	9.7	TGGAGGCcaa**ATG**		N/A			
7	+	4832	5077	81	9.4	4.32	AGGCGGGttggtt**GTG**		N/A			
8	+	5087	5260	57	6.7	9.25	AGGAGTAttaa**ATG**		gp07 [*Enterobacteria* phage phiEcoM-GJ1] (56/57; 98%)	57	6 × 10^−34^	YP_001595402.1
9	+	5356	5667	103	11.6	4.5	AGGTAATtaa**ATG**		gp08 [*Enterobacteria* phage phiEcoM-GJ1] (84/99; 85%)	99	2 × 10^−54^	YP_001595404.1
10	+	5683	5943	86	9.7	5.6	GGGAGTTatt**ATG**		gp09 [*Enterobacteria* phage phiEcoM-GJ1] (79/86; 92%)	87	6 × 10^−53^	YP_001595404.1
11	+	5936	6118	60	6.4	4.64	TGGGAGTtctgtacc**ATG**		N/A			
12	+	6121	6348	75	8.4	5.24	AGGATAAtc**ATG**		gp10 [*Enterobacteria* phage phiEcoM-GJ1] (67/75; 89%)	75	5 × 10^−45^	YP_001595405.1
13	+	6345	6575	76	8.6	9.58	ACAAGGTttattgca**ATG**		gp11 [*Enterobacteria* phage phiEcoM-GJ1] (42/68; 62%)	68	1 × 10^−16^	YP_001595406.1
14	+	6639	6929	96	10.7	9.47	TGGAGCAttt**ATG**		gp12 [*Enterobacteria* phage phiEcoM-GJ1] (87/96; 91%)	96	7 × 10^−59^	YP_001595407.1
15	+	6922	7155	77	8.8	5.22	AGAAGGTgaagc**GTG**		gp13 [*Enterobacteria* phage phiEcoM-GJ1] (68/77; 88%)	77	5 × 10^−44^	YP_001595408.1
16	+	7152	7439	95	10.8	9.3	TGGAGAAattaaagca**ATG**		gp14 [*Enterobacteria* phage phiEcoM-GJ1] (84/95; 88%)	95	1 × 10^−54^	YP_001595409.1
17	+	7439	7636	65	7.82	9.81	AGGTGATgta**ATG**		IME11_76 [Escherichia phage IME11] (29/65;45%)	68	3 × 10^−7^	YP_006990681.1
18	+	7715	8197	160	18.8	8.79	TGGAGGGctt**ATG**		CBB_348 [Pectobacterium phage CBB] (72/160; 45%)	161	2 × 10^−36^	AMM43911.1
19	+	8323	8700	125	14.2	5.78	AAGAGAAtcttaatc**ATG**	ssDNA-binding protein	gp15 [*Enterobacteria* phage phiEcoM-GJ1] (96/125; 77%)	126	6 × 10^−54^	YP_001595410.1
20	+	8823	9719	298	33.2	7.74	GTGAGGAatatc**ATG**		gp17 [*Enterobacteria* phage phiEcoM-GJ1] (159/229; 69%)	229	5 × 10^−109^	YP_001595412.1
21	+	9775	10,125	116	13.1	6.07	CGGAGCAttt**ATG**		gp18 [*Enterobacteria* phage phiEcoM-GJ1] (114/116; 98%)	116	2 × 10^−80^	YP_001595413.1
22	+	10,122	10,355	77	8.72	5.29	AGGAAGTtaa**ATG**		gp19 [*Enterobacteria* phage phiEcoM-GJ1] (56/77; 73%)	77	5 × 10^−32^	YP_001595414.1
23	+	10,345	10,614	89	10.3	4.7	AGGAAATccattcc**GTG**		N/A			
24	+	10,607	11,023	138	15.7	9.48	AGGAGCTgaaaa**ATG**	endolysin	gp21 [*Enterobacteria* phage phiEcoM-GJ1] (110/131; 84%)	131	1 × 10^−72^	YP_001595416.1
25	+	11,044	11,322	92	10.9	5.7	TGGAGCAtccg**ATG**		N/A			
26	+	11,309	11,857	182	21.2	4.03	GGGAGAAactca**ATG**	antirestriction protein	gp22 [*Enterobacteria* phage phiEcoM-GJ1] (145/181; 80%)	181	2 × 10^−100^	YP_001595417.1
27	+	11,850	12,089	79	9.2	6.9	CGAAGGGatactattctcaa**ATG**		gp23 [*Enterobacteria* phage phiEcoM-GJ1] (73/79; 92%)	79	4 × 10^−48^	YP_001595418.1
28	+	13,092	13,295	67	7.5	4.58	TGGAGAGttcct**ATG**		gp25 [*Enterobacteria* phage phiEcoM-GJ1] (57/67; 85%)	69	3 × 10^−33^	YP_001595420.1
29	+	13,292	13,582	96	11.3	9.1	AGGAGCTgcaaaa**ATG**		N/A			
30	+	13,579	13,869	96	10.6	5.25	CGGAGTTccatt**TTG**		gp27 [*Enterobacteria* phage phiEcoM-GJ1] (93/96; 97%)	97	3 × 10^−59^	YP_001595422.1
31	+	13,872	14,546	224	25.9	8.28	ACAAGGCcactaaaa**ATG**		gp28 [*Enterobacteria* phage phiEcoM-GJ1] (223/224; 99%)	224	2 × 10^−165^	YP_001595423.1
32	+	14,670	15,008	112	12.3	4.47	ATAAGGTatatacaa**ATG**		gp29 [*Enterobacteria* phage phiEcoM-GJ1] (111/112; 99%)	112	9 × 10^−75^	YP_001595424.1
33	+	15,395	15,532	45	5.1	8.99	CGGAGCAataattaat**TTG**		N/A			
34	+	15,547	15,789	80	8.8	9.24	AGAAGCTatgccaat**GTG**		gp30 [*Enterobacteria* phage phiEcoM-GJ1] (77/80; 96%)	80	1 × 10^−50^	YP_001595425.1
35	+	16,029	16,169	46	4.8	3.76	TGGAGTCctc**ATG**		N/A			
36	+	16,178	16,414	78	8.5	9.05	AGGTGATtt**ATG**		gp31 [*Enterobacteria* phage phiEcoM-GJ1] (77/78; 99%)	78	1 × 10^−44^	YP_001595426.1
37	+	17,404	17,634	76	8.5	4.89	TGGAGAGaaac**ATG**		gp32 [*Enterobacteria* phage phiEcoM-GJ1] (74/76; 97%)	76	2 × 10^−44^	YP_001595427.1
38	+	17,691	18,341	216	24.8	5.99	CGGAGAGcaa**ATG**	thymidylate synthase	gp33 [*Enterobacteria* phage phiEcoM-GJ1] (196/216; 91%)	216	8 × 10^−149^	YP_001595428.1
39	+	18,346	20,109	587	66	5.95	ACCAGGAataaataa**ATG**	helicase/primase	gp35 [*Enterobacteria* phage phiEcoM-GJ1] (560/587; 95%)	587	0	YP_001595430.1
40	+	20,175	22,109	644	74.6	6.41	TGGAGCCatact**GTG**	DNA polymerase	gp37 [*Enterobacteria* phage phiEcoM-GJ1] (637/644; 99%)	644	0	YP_001595432.1
41	+	22,109	22,381	90	10.1	4.37	CAGAGATtcacta**ATG**		gp38 [*Enterobacteria* phage phiEcoM-GJ1] (86/90; 96%)	90	2 × 10^−54^	YP_001595433.1
42	+	22,412	23,278	288	31	4.86	AGGTACTcaaa**ATG**		gp39 [*Enterobacteria* phage phiEcoM-GJ1] (288/288; 100%)	288	0	YP_001595434.1
43	+	23,312	24,349	345	39.4	8.09	GGGAGCCtttaatt**TTG**	exonuclease	gp40 [*Enterobacteria* phage phiEcoM-GJ1] (342/345; 99%)	345	0	YP_001595435.1
44	+	24,361	24,885	174	20.1	9.46	TGGAGTTgga**ATG**		gp41 [*Enterobacteria* phage phiEcoM-GJ1] (173/174; 99%)	174	3 × 10^−124^	YP_001595436.1
45	+	24,875	25,630	251	28.5	8.64	AGAAAGAatctta**ATG**	DNA ligase	gp42 [*Enterobacteria* phage phiEcoM-GJ1] (233/251; 93%)	251	6 × 10^−175^	YP_001595437.1
46	+	25,623	26,249	208	23.5	6.38	GTGAGGAaagtt**TTG**		gp43 [*Enterobacteria* phage phiEcoM-GJ1] (203/208; 98%)	208	1 × 10^−147^	YP_001595438.1
47	+	26,252	26,839	195	20.7	6.66	ATCAAGTagagaaataatc**ATG**	deoxyuridine 5’-triphosphate nucleotidylhydrolase	gp44 [*Enterobacteria* phage phiEcoM-GJ1] (164/195; 82%)	199	4 × 10^−105^	YP_001595439.1
48	+	26,858	27,064	68	8.2	4.32	TGGAGCAtcc**ATG**		PP74_27 [Pectobacterium phage PP74] (37/68; 54%)	73	3 × 10^−17^	APD19639.1
49	+	27,082	27,411	109	12.2	9.7	TGGAACCtatctgaa**ATG**		gp45 [*Enterobacteria* phage phiEcoM-GJ1] (109/109; 100%)	109	4 × 10^−74^	YP_001595440.1
50	+	27,467	27,652	61	7	4.4	CGGAGTCgctt**ATG**		gp46 [*Enterobacteria* phage phiEcoM-GJ1] (61/61; 100%)	61	1 × 10^−37^	YP_001595441.1
51	+	27,672	29,690	672	76	6.02	AAGAGAAcgaatca**ATG**	large subunit terminase	gp48 [*Enterobacteria* phage phiEcoM-GJ1] (667/671; 99%)	671	0	YP_001595443.1
52	+	29,693	29,905	70	7.9	9.18	TGGATGTaaat**ATG**		gp49 [*Enterobacteria* phage phiEcoM-GJ1] (70/70; 100%)	70	7 × 10^−43^	YP_001595444.1
53	+	29,905	31,221	438	49.1	8.16	AGGAAGAaata**ATG**	portal protein	gp50 [*Enterobacteria* phage phiEcoM-GJ1] (434/438; 99%)	438	0	YP_001595445.1
54	+	31,190	32,254	354	39	4.8	AAAGGGTaacgcaa**GTG**		gp51 [*Enterobacteria* phage phiEcoM-GJ1] (353/354; 99%)	354	0	YP_001595446.1
55	+	32,264	32,737	157	16.4	6.26	ATAAGGTaagaca**ATG**		gp52 [*Enterobacteria* phage phiEcoM-GJ1] (147/157; 94%)	157	2 × 10^−101^	YP_001595447.1
56	+	32,818	33,030	70	7.3	6.06	TGTAACT**GTG**		gp67 [Erwinia phage vB_EamM-Y2] (51/70; 73%)	90	2 × 10^−23^	YP_007004717.1
57	+	33,086	34,093	335	36.7	5.17	TGGATTAaattac**ATG**	major capsid protein	gp53 [*Enterobacteria* phage phiEcoM-GJ1] (323/335; 96%)	335	0	YP_001595448.1
58	+	34,140	34,580	146	16.1	5.34	AAGAGAAatagta**ATG**		gp54 [*Enterobacteria* phage phiEcoM-GJ1] (116/146; 79%)	146	1 × 10^−71^	YP_001595449.1
59	+	34,581	34,970	129	14.6	4.48	AGTTGGCgtaa**ATG**		gp55 [*Enterobacteria* phage phiEcoM-GJ1] (124/129; 96%)	129	2 × 10^−86^	YP_001595450.1
60	+	34,967	35,329	120	13.9	9.16	GGGTCACagtt**TTG**		gp56 [*Enterobacteria* phage phiEcoM-GJ1] (120/120; 100%)	120	4 × 10^−85^	YP_001595451.1
61	+	35,326	35,838	170	19.3	4.98	AGGAGTTagagaa**ATG**		gp57 [*Enterobacteria* phage phiEcoM-GJ1] (167/170; 98%)	170	2 × 10^−120^	YP_001595452.1
62	+	35,839	37,287	482	50.9	4.75	AGGGAATctaa**ATG**		gp58 [*Enterobacteria* phage phiEcoM-GJ1] (447/482; 93%)	482	0	YP_001595453.1
63	+	37,298	37,753	151	16.5	6.55	AGGTGCGataa**GTG**		gp59 [*Enterobacteria* phage phiEcoM-GJ1] (148/151; 98%)	151	2 × 10^−104^	YP_001595454.1
64	+	37,765	38,223	152	17.3	5.1	AGTAAGT**ATG**		gp60 [*Enterobacteria* phage phiEcoM-GJ1] (152/152; 100%)	152	4 × 10^−107^	YP_001595455.1
65	+	38,229	38,399	56	6.7	4.67	CGGAGACagtttagtatcc**ATG**		gp61 [*Enterobacteria* phage phiEcoM-GJ1] (55/56; 98%)	73	8 × 10^−32^	YP_001595456.1
66	+	38,383	42,108	1241	134.6	5.36	AGAAACTcgaaccagtag**ATG**	tail fiber	gp62 [*Enterobacteria* phage phiEcoM-GJ1] (946/1239; 76%)	1239	0	YP_001595457.1
67	+	42,182	43,291	369	40.7	5.13	AATAGGTatatcgca**ATG**		gp63 [*Enterobacteria* phage phiEcoM-GJ1] (366/369; 99%)	369	0	YP_001595458.1
68	+	43,291	44,178	295	31.2	5.98	TGGAGTCatttta**ATG**		gp64 [*Enterobacteria* phage phiEcoM-GJ1] (295/295; 100%)	295	0	YP_001595459.1
69	+	44,175	44,537	120	13.7	5.07	GGGACGTatcct**ATG**		gp65 [*Enterobacteria* phage phiEcoM-GJ1] (120/120; 100%)	120	2 × 10^−84^	YP_001595460.1
70	+	44,530	45,324	264	28.2	5.8	AGAGTGTacttgaac**GTG**	baseplate assembly protein	gp66 [*Enterobacteria* phage phiEcoM-GJ1] (263/264; 99%)	264	0	YP_001595461.1
71	+	45,324	45,695	123	13.5	5.22	ATGAAATa**ATG**		gp67 [*Enterobacteria* phage phiEcoM-GJ1] (117/123; 95%)	123	7 × 10^−80^	YP_001595462.1
72	+	45,671	46,828	385	41.2	4.55	CGGAATTcttaac**ATG**		gp68 [*Enterobacteria* phage phiEcoM-GJ1] (361/385; 94%)	385	0	YP_001595463.1
73	+	46,830	47,471	213	23.5	5.82	CAGATGTgacagtataat**ATG**		gp69 [*Enterobacteria* phage phiEcoM-GJ1] (193/213; 91%)	213	1 × 10^−139^	YP_001595464.1
74	+	47,471	48,619	382	42.3	5.49	CGGAGAAata**ATG**		gp70 [*Enterobacteria* phage phiEcoM-GJ1] (342/382; 90%)	382	0	YP_001595465.1
75	+	48,619	50,016	465	50.2	8.17	AGGCCATa**ATG**		gp71 [*Enterobacteria* phage phiEcoM-GJ1] (314/465; 68%)	465	0	YP_001595466.1
76	+	50,025	51,071	348	36.3	6.6	AGGATTCaaa**ATG**	tail fiber protein	gp72 [*Enterobacteria* phage phiEcoM-GJ1] (250/348; 72%)	356	3 × 10^−156^	YP_001595467.1
77	+	51,079	51,420	113	12.3	7.95	AGGAACTc**ATG**	holin	gp73 [*Enterobacteria* phage phiEcoM-GJ1] (110/113; 97%)	113	8 × 10^−74^	YP_001595468.1
78	+	51,438	51,992	184	20.7	9.57	AGGAACTcga**ATG**	endolysin	gp74 [*Enterobacteria* phage phiEcoM-GJ1] (178/184; 97%)	184	7 × 10^−131^	YP_001595469.1
79	+	51,992	53,092	366	42.2	4.76	AGGAAATctgta**ATG**	ribonucleotide reductase beta subunit	gp75 [*Enterobacteria* phage phiEcoM-GJ1] (340/366; 93%)	372	0	YP_001595470.1

^a^ Start codon indicated in bold; Match to SD sequence is indicated by underlining; SD position is indicated in uppercase. ^b^ The number of identical amino acids/The total of amino acids of smallest protein.
